# The outcome of COVID-19 among the geriatric age group in African countries: protocol for a systematic review and meta-analysis

**DOI:** 10.1186/s40733-020-00064-8

**Published:** 2020-10-08

**Authors:** Degena Bahrey Tadesse, Shishay Wahdey, Melaku Negash, Ebud Ayele, Teklehaimanot Gereziher Haile, Kbrom Gemechu Kiros, Yohannes Ashebir Tesfamichael, Kiros Belay Gebrekidan

**Affiliations:** 1 Department of Adult Health Nursing, School of Nursing, Aksum University, Aksum, Ethiopia; 2 Department of Reproductive Health, School of Public Health, Mekelle University, Mekelle, Ethiopia; 3 Department of Reproductive Health, School of Public Health, Aksum University, Aksum, Ethiopia; 4 Department of Maternal and Neonatal Nursing, School of Nursing, Aksum University, Aksum, Ethiopia; 5 Department of Adult Health Nursing, School of Nursing, Adigrat University, Adigrat, Ethiopia; 6 Department of Pediatrics and Child Health Nursing, School of Nursing, Adigrat University, Adigrat, Ethiopia; 7 Department of Adult Health Nursing, School of Nursing, Mekelle University, Mekelle, Ethiopia

**Keywords:** Africa, COVID-19, Geriatric, Outcome

## Abstract

**Background:**

According to the World Health Organization (WHO), the outbreak of coronavirus disease in 2019 (COVID-19) has been declared as a pandemic and public health emergency that infected more than 5 million people worldwide at the time of writing this protocol. Strong evidence for the outcome of COVID-19 among the geriatric age group has not been published in Africa. Therefore, this protocol will be served as a guideline to conduct a systematic review and meta-analysis of the outcome of COVID-19 among the geriatric age group in Africa.

**Methods:**

Published and unpublished studies on the outcome of COVID-19 among the geriatric age group in Africa and written in any language will be included. Databases (PubMed / MEDLINE, Google Scholar, Google, EMBASE, Web of Science, Microsoft Academic, WHO COVID-19 database, Cochran Library, Africa Wide Knowledge, and Africa Index Medicus) from March to August 2020 will be searched. Two independent reviewers will select, screen, extract data, and assess the risk of bias. The proportion will be measured using a random-effects model. Subgroup analysis will be conducted to manage heterogeneity. The presence of publication bias will be assessed using Egger’s test and visual inspection of the funnel plots. This systematic and meta-analysis review protocol will be reported per the PRISMA-P guidelines.

**Conclusion:**

This systematic review and meta-analysis protocol will be expected to quantify the outcome of COVID-19 among the geriatric age group in Africa.

**Systematic review registration:**

This protocol was submitted for registration with the International Prospective Register of Systematic Reviews (PROSPERO) in April 2020 and accepted with the registration number: (https://www.crd.york.ac.uk/PROSPERO). CRD42020180600.

## Background

Coronavirus disease 2019 (abbreviated “COVID-19”) is an infectious respiratory illness caused by a novel coronavirus, first identified in Wuhan, China, in December 2019. It is a large class of viruses that have been relatively widespread all across the world. The virus has low pathogenicity and high transmissibility capacity [1]. The Chinese authorities in Wuhan City, the capital of the province of Hubei, China, first announced this at the end of December 2019 [2–5]. After originating in Wuhan China last December 2019, COVID-19 has spread to at least 200 countries and region. The coronavirus disease 2019 (COVID-19) outbreak was declared a public health emergency of international concern by the World Health Organization (WHO) [6]. According to the WHO, the outbreak of coronavirus disease in 2019 (COVID-19) has been a pandemic that infected more than 5 million people at the time of writing this protocol and caused more than 324,000 deaths and 1.7 million recoveries worldwide within the 6 months duration [7]. Of the total cases, 15% develop severe disease, including pneumonia, and 5% become critically ill with respiratory failure, septic shock, and/or multi-organ failure [8].

The geriatric age groups were the most vulnerable age group and the Wuhan virus killing mostly the elderly. Of the 70- to 79-year-olds infected with coronavirus there, 8% have died. Among the over-80s, the case fatality rate is 15% [9].

A modeling study in Africa, classifying African countries as having high risk with 13 top WHO highest priority (Egypt, Algeria, and South Africa), moderate risk (Nigeria, Ethiopia, Morocco, Sudan, Angola, Tanzania, Ghana, and Kenya) and all other countries had low to moderate importation risk. The fatality rate of COVID in north African countries expected to be 11.02% [10, 11].

Generally speaking, African countries have poor health systems and this remains a source of concern, particularly in the event of an increase in outbreaks [12]. Due to this reason at the end of the pandemic, Africa might have some of the worst consequences of this COVID-19 pandemic.

In the WHO Africa region alone 83,000 to 190, 000 people could die of COVID-19 and 29 million to 44 million could get infected in the first year of the pandemic if nt measurement will not be taken. The research, which is based on prediction modeling, looks at 47 countries in the WHO African Region with a total population of 1 million. There would be an estimation of 3.6 to 5.5 million COVID-19 hospitalizations, of those 82,000 to 167,000 would be severe cases requiring oxygen, and 52,000 to 107,000 would be critical cases requiring breathing support [13].

There were different reports regarding the COVID-19 [14–16] but; there are no pooled results regarding the outcome of COVID-19 among the geriatric age group in Africa. Therefore, this study protocol will be guided to conduct a systematic review and meta-analysis of the outcome of COVID-19 among the geriatric age group in Africa.

## Methods

### Protocol registration

This review is registered in PROSPERO International Prospective Register of Systematic Reviews (CRD42020180600**)** (https://www.crd.york.ac.uk/PROSPERO)) and reported according to Preferred Reporting Items for Systematic reviews and MetaAnalysis protocol (PRISMA-P) guidelines [17] (Table 1).

**Table 1:** PRISMA-P (Preferred Reporting Items for Systematic Review and Meta-Analysis Protocols) 2015 checklist: recommended items to address in a systematic review protocol

Section/topic	Item No	Checklist item	Information reported		Line number(s)
			Yes	No	
Administrative Information					
Title:					
Identification	1a	Identify the report as a protocol of a systematic review			
Update	1b	If the protocol is for an update of a previous systematic review, identify as such			
Registration	2	If registered, provide the name of the registry (e.g., PROSPERO) and registration number in the Abstract			
Authors					
Contact	3a	Provide name, institutional affiliation, and e-mail address of all protocol authors; provide the physical mailing address of the corresponding author			
Contributions	3b	Describe contributions of protocol authors and identify the guarantor of the review			
Amendments	4	If the protocol represents an amendment of a previously completed or published protocol, identify as such and list changes; otherwise, state plan for documenting important protocol amendments			
Support					
Sources	5a	Indicate sources of financial or other support for the review			
Sponsor	5b	Provide a name for the review funder and/or sponsor			
Role of sponsor/funder	5c	Describe roles of funder(s), sponsor(s), and/or institution(s), if any, in developing the protocol			
Introduction					
Rationale	6	Describe the rationale for the review in the context of what is already known			
Objectives	7	Provide an explicit statement of the question(s) the review will address concerning participants, interventions, comparators, and outcomes (PICO)			
Methods					
Eligibility criteria	8	Specify the study characteristics (e.g., PICO, study design, setting, time frame) and report characteristics (e.g., years considered, language, publication status) to be used as criteria for eligibility for the review			
Information sources	9	Describe all intended information sources (e.g., electronic databases, contact with study authors, trial registers, or other grey literature sources) with planned dates of coverage			
Search strategy	10	The present draft of the search strategy to be used for at least one electronic database, including planned limits, such that it could be repeated			
Study Records					
Data management	11a	Describe the mechanism(s) that will be used to manage records and data throughout the review			
Selection process	11b	State the process that will be used for selecting studies (e.g., two independent reviewers) through each phase of the review (i.e., screening, eligibility, and inclusion in meta-analysis)			
Data collection process	11c	Describe the planned method of extracting data from reports (e.g., piloting forms, done independently, in duplicate), and processes for obtaining and confirming data from investigators			
Data items	12	List and define all variables for which data will be sought (e.g., PICO items, funding sources), any pre-planned data assumptions and simplifications			
Outcomes and prioritization	13	List and define all outcomes for which data will be sought, including prioritization of main and additional outcomes, with rationale			
Risk of bias in individual studies	14	Describe anticipated methods for assessing the risk of bias of individual studies, including whether this will be done at the outcome or study level, or both; state how this information will be used in data synthesis			
*Data*					
Synthesis	15a	Describe criteria under which study data will be quantitatively synthesized			
	15b	If data are appropriate for quantitative synthesis, describe planned summary measures, methods of handling data, and methods of combining data from studies, including any planned exploration of consistency (e.g., *I* ^2^, Kendall’s tau)			
	15c	Describe any proposed additional analyses (e.g., sensitivity or subgroup analyses, meta-regression)			
	15d	If quantitative synthesis is not appropriate, describe the type of summary planned			
Meta-bias(es)	16	Specify any planned assessment of meta-bias(es) (e.g., publication bias across studies, selective reporting within studies)			
Confidence in cumulative evidence	17	Describe how the strength of the body of evidence will be assessed (e.g., GRADE)			

### Search strategy and data extraction

The search strategy has been applied using Online Databases (PubMed / MEDLINE, Google Scholar, Google, EMBASE, Web of Science, Microsoft Academic, WHO COVID-19 database, Cochran Library, Africa Wide Knowledge, and Africa Index Medicus) from March to August 2020(Table 2). During the PROSPERO registration, a total of 10 articles were identified. The search terms which shall be used: “Wuhan coronavirus” OR “COVID-19” OR “novel coronavirus” OR “2019-nCoV” OR “coronavirus disease” OR “SARS-CoV-2” OR “SARS2” OR “severe acute respiratory syndrome coronavirus 2” OR” admission” OR” Burden” OR “Outcome”. Other searching terms will be used “mortality” OR “prevalence” OR” incidence” OR” cardiovascular complications” OR” Geriatrics Age Group” OR” renal complications” “hematological complications of COVID-19” OR”prevalence of asymptomatic, mild, moderate and severe cases” OR “admission” (“number admitted to specialized units or intensive care units” OR” outcome” (“number of infected patients OR” “number of recoveries” OR” case fatality rate” OR “number of cured patients readmitted” OR “long term complications” such as chronic heart failure, cardiac arrhythmias, and recurrent thromboembolic diseases.

**Table 2:** Searching strategy

Serial number	Databases	Number of the article found	Number of the article included	Number of Excluded article	Reason for exclusion
1	PubMed	n=	n=	n=	
2	Google Scholar	n=	n=	n=	
3	Web of Science	n=	n=	n=	
4	Cochran Library	n=	n=	n=	
5	Africa Wide Knowledge	n=	n=	n=	
6	Africa Index Medicus	n=	n=	n=	
7	Microsoft Academic	n=	n=	n=	
8	WHO COVID-19 database	n=	n=	n=	
9	Unpublished (pre-prent, manuscript, thesis and report from WHO,CDC)	n=	n=	n=	

Searching results will be independently evaluated by two different reviewers. The literature search technique will be developed using the headings of the medical subject headings (Mesh), BOOLEAN (AND/OR) operator will be used.

The blinding will be maintained by using the Royyan that allows/ obligates each reviewer to work without knowing the other reviewer’s choice. This review will be created using the metadata of the “COVID-19 Open Research Dataset” (https://pages.semanticscholar.org/coronavirus-research) (updated May 2020). We only uploaded the metadata (reference) file of 63 k + coronavirus and COVID-19 research articles with links to PubMed, Microsoft Academic, and the WHO COVID-19 database of publications. We had to transform the metadata file (using https://github.com/rayyanqcri/CORD-19-importer) to make it compatible with Rayyan. r.

### Selection and data collection process

Data will be Extracted using the predefined standardized extraction form. Full texts for the eligible titles and/or abstracts including those where there is uncertainty will be obtained for further assessment on whether to include in the study or not. Where necessary, authors will be contacted for additional information to confirm eligibility of studies. The agreement between review authors will be measured using Cohen’s κ statistic. Reasons for excluding articles will be recorded.

Where there is missing information, the corresponding author of the study will be contacted to request the missing information. A maximum of three emails will be sent to the corresponding author to request for additional information before excluding the study. For studies appearing in more than one published article, we will consider the most recent, comprehensive, and with the largest sample size. For surveys appearing in one article with multiple surveys conducted at different time points, we shall treat each survey as a separate study. For multi-national studies, data will be separated to show the estimate at the country level.

Data extracted comprised information about the month of publication, country, and design of the study, admission rate, burden, outcome, diagnostic criteria, comorbidity, COVID-19, mean age,ethnicity, sex (male proportion), signs and symptoms, complications, prevalence and/or incidence, and risk factors.

### Inclusion and exclusion criteria

Studies presented as original articles studies that assessed outcomes from COVID-19 will be included.

#### Types of studies

Observational studies (including cross-sectional, case-control, and cohort) and the randomized controlled trial will be included. In the case of duplicate reports, the most comprehensive and up-to-date version will be taken into account.

#### Participants

All Patients from 65 years old or more, who are African residence and will be diagnosed as having COVID-19.

#### Intervention(s)/exposure(s)

Demographic, clinical, laboratory, management, and outcome data will be reviewed.

#### Outcome

Epidemiological data, mortality, and clinical outcomes of COVID-19. Establishing the clinical and epidemiological features, outcomes of COVID-19.

#### Settings

Hospital-based studies.

#### Publication date

March to August 2020.

#### Language

All published and unpublished papers (thesis, manuscript, pre-print pending to be published, the report from WHO, uCommunicable Diseases Control, United Nations, and health authorities in different African countries) without the restriction of language will include in our review.

#### Method of diagnosis

No restriction on methods of diagnosis but we will conduct subgroup analysis based on diagnostic tools. WHO interim guidance and /or any WHO-recommended diagnostic criteria will be considered [18] (Table 3).

**Table 3:** Laboratory testing for coronavirus disease (COVID-19) in suspected human cases: interim guidance

Test	Type of sample	Timing
Nucleic acid amplification tests (NAAT)	**Lower respiratory tract**	Collect on presentation. Possibly repeated sampling to monitor clearance. Further research needed to determine effectiveness and reliability of repeated sampling.
	Sputum	
	Aspirate	
	Lavage	
	**Upper respiratory tract**	
	Nasopharyngeal and Oropharyngeal swabs Nasopharyngeal wash/nasopharyngeal aspirate.	
Serology	Serum for serological testing once validated and available.	Paired samples are necessary for confirmation with the initial sample collected in the first week of illness and the second ideally collected 2–4 weeks later (optimal timing for convalescent sample needs to be established).

#### Exclusion criteria

Studies that did not explain the criteria for the level COVID-19 outcome; studies that didn’t state the number of patients with COVID-19 will be excluded. Studies not performed in humans, qualitative studies, studies that lack relevant data needed to compute the outcome will be excluded. Experimental studies, letters, reviews, commentaries, editorials, case reports, or case series will be not included.

### Quality assessment and risk of bias in individual studies

To assess the risk of bias and quality of studies included in this review, a tool developed by Hoy et al. for prevalence studies will be used [19] The tool contains 11 items; items 1–4 assess the external validity, 5–10 assess the internal validity, and item 11 is a summary of the overall risk by the reviewer based on the responses of the above 10 items which are scored 1 if yes and 0 if no. Studies will be classified as having a low (> 8), moderate or high (≤ 5) risk of bias. Additional file 1 shows this in more detail regarding the checklist of bias measurement on the observational study. For RCTs, we will use the SPIRIT 2013 Checklist [20]. Additional file 2 shows this in more detail regarding the checklist of SPIRIT.

### Data management

A tool has been developed a priori to conduct the searching strategy and this will help to guide the screening and selection process. The tool will be piloted and revised before data extraction begins. First the search results will be uploaded to EndNote software and then duplicates will be removed.

### Data items

The extraction form will be includes data on general information, authors, month, country, and region, type of publication, study characteristics (study design, setting, sample size, response rate, mean or median age, or age range), the outcome.

### Outcomes

The outcome of COVID − 19 among the geriatric age group in Africa.

### Data synthesis, analysis, and presentation

The data will be analyzed using the R software; V.3.5.3. All analyses will be performed using a “metaprop” routine using R version 3.5.3 for Windows [18]. Results will be reported as proportions with corresponding 95% confidence intervals (CIs). Forest plots will be drawn to visualize the combined outcome of COVID-19 and the extent of statistical heterogeneity between studies. Statistical heterogeneity will be assessed using theχ2 test on Cochrane’s Q statistic, 20 and quantified by calculating the I^2^ statistic (with values of 25, 50, and 75% is representative of the low, medium, and high heterogeneity, respectively) [19]. There will be a clinical heterogeneity between studies included in this study. The random-effects will be used to estimate the overall pooled outcome of COVID-19 in Africa. The presence of publication bias will be assessed using Egger’s test and funnel plots [20]. *P*-value < 0.10 on the Egger’s test will be considered statistically significant for publication bias. Moreover, other relevant findings will be summarized in a narrative format.

The crude numerators and denominators from the individual studies will be used to recalculate the study-specific outcome of COVID-19. The outcome estimates will be summarized by African geographic regions.

A meta-analysis will be performed on variables that are similar across the included studies.

A subgroup analyses will be performed based on the countries where the study conducted, diagnostic method they used, based on their ethnic background (African origin and -non-African ethnic origin). The inter-rater agreements between the researchers involved in study selection and those involved in the identification of risk of bias will be assessed using *κ* Cohen’s coefficient.

## Discussion

**Fig. 1:**
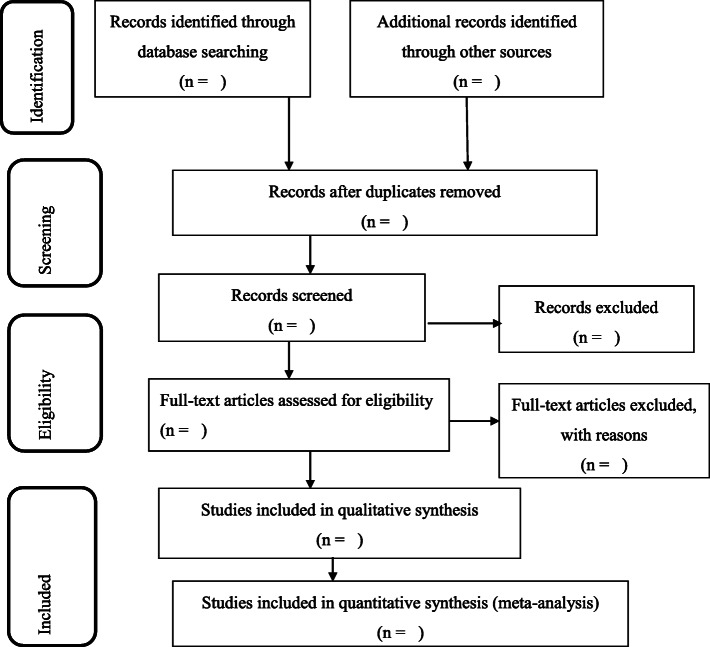
Flow chart diagram will be shown the selection of articles for systemic review and meta-analysis of the outcome of COVID-19 among the geriatric age group in Africa

This systematic review and meta-analysis will be presented per the PRISMA checklist guidelines [14]. The PRISMA flow diagram will be used to record the different phases of the review process [14] (Fig. 1).

The summary will be used to display the data on the distribution of COVID-19 by variables of interest such as gender, residence, setting, and person-level characteristics. Funnel plots will be used to visualize publication bias of the included studies. Forest plots will be used to estimates the outcome of COVID-19 among the geriatric age group in Africa for the included studies as an overall pooled estimate for Africa. Results from this review will inform healthcare providers on the outcome of COVID − 19, hence providing evidence will bring the required changes needed in clinical practice and will support healthcare services in line with patients’ needs. Findings from this review will be shared in conferences, peer-review journals, and social media platforms.

## Conclusion

This systematic review and meta-analysis will be expected to quantify the outcome of COVID-19 among the geriatric age group in Africa to guide policies and interventions.

## Supplementary information

**Additional file 1.** Quality assessment checklist for prevalence studies (adapted from Hoy et al).

**Additional file 2.** SPIRIT 2013 Checklist: Recommended items to address in a randomized controlled trial.

## Data Availability

The datasets used and/or analyses during the study will be presented within the manuscript and available from the corresponding author on reasonable request.

## Abbreviations

PRISMA-P: Preferred Reporting Items for Systematic reviews and Meta-Analysis protocol
WHO: World Health Organization
COVID-19: Coronavirus Disease in 2019
